# The information needs of people with degenerative cervical myelopathy: A qualitative study to inform patient education in clinical practice

**DOI:** 10.1371/journal.pone.0285334

**Published:** 2023-05-19

**Authors:** Irina Sangeorzan, Panoraia Andriopoulou, Benjamin M. Davies, Angus McNair

**Affiliations:** 1 Myelopathy.org, Cambridge, Cambridgeshire, United Kingdom; 2 School of Humanities, Hellenic Open University, Patras, Greece; 3 Academic Neurosurgery Unit, Department of Clinical Neurosurgery, University of Cambridge, Cambridge, Cambridgeshire, United Kingdom; 4 Centre for Surgical Research, Bristol Medical School, University of Bristol, United Kingdom; The University of York, UNITED KINGDOM

## Abstract

**Background:**

Individuals with lifelong illnesses need access to adequate information about their condition to make optimal health decisions. Degenerative Cervical Myelopathy (DCM) is the most common form of spinal cord dysfunction in adults worldwide. Its chronic and debilitating nature, varied impact, clinical trajectory, and management options necessitate appropriate informational support to sustain effective clinical and self-directed care strategies. However, before clinicians can meet patients’ information needs, they must first have an understanding of their baseline requirements. This study explores the information needs of people with DCM (PwCM). In doing so, it provides a starting point for the development of patient education and knowledge management strategies in clinical practice.

**Methods:**

Semi-structured interviews with PwCM were conducted using an interview guide. Interviews were audio-recorded and transcribed verbatim. Thematic analysis according to Braun and Clarke’s six-phase approach was used to analyse the data. Findings were reported according to the Consolidated Criteria for Reporting Qualitative Research (COREQ) guidelines.

**Results:**

Twenty PwCM (65% female, 35% male), with ages ranging from 39 to 74 years old participated in the interviews. The findings indicated that the provision of information to PwCM during clinical interactions varies. Accordingly, PwCM’s information needs were broad-ranging, as was the nature of the information they found useful. Three main themes were identified (1) Variation in the provision of information to PwCM during clinical interactions, (2) Variations in the information needs of PwCM, and (3) Information that PwCM find useful.

**Conclusion:**

Efforts must turn to adequately educating patients at the time of the clinical encounter. A comprehensive and consistent patient-centered information exchange in DCM is necessary to achieve this.

## Introduction

Adequate provision of information by healthcare professionals that is material to and aligned with patients’ informational needs is a mainstay of patient-centred care [1–6]. Informational support is particularly important in the context of unresolved illnesses such as chronic illness or disability, which require ongoing management on a long-term basis [7]. Indeed, the progressive nature of chronic conditions sees patients engaging in multi-faceted, preference- and context-sensitive health management judgements many of which are characterised by varied clinical outcomes [8, 9]. Moreover, initial health decisions often need frequent revision throughout an individual’s lifetime [10, 11]. Quality care for chronic illness, therefore, requires a personalised approach [12]. That is, in order for care decisions to be optimal they must first be centered on patients’ circumstances, values and priorities [13–16]. Effective informational support is vital to that end [13].

Unsurprisingly, demand for information among chronic illness patients is generally high, its provision increasingly important as the management of the condition expands in complexity [7]. Despite this, practitioners often underestimate patients’ desire for information [17–20] or misperceive the efficacy of information transfer [18]. For patients to express a preference or value-based consideration, they must first have an adequate and sufficient understanding of their circumstances [21]. Understanding and meeting patients’ information needs, therefore, is essential if clinicians are to guide optimal health outcomes [22–26]. Mounting evidence from the realm of chronic illnesses points to improved health outcomes when patients are equipped with up-to-date information that is relevant to their circumstances [22, 27–30].

Patient education is therefore likely highly relevant to DCM [31], a chronic neurological condition that arises due to age-related degenerative changes in the surrounding structures of the cervical spine [32], as care is highly context- and individual-dependant. Throughout the trajectory of the illness, PwCM [33] are confronted with care decisions that are highly contingent upon individual, contextual and technical factors [34–36]. Variations in clinical presentation, severity, multiple treatment options, and the inherent recurrent nature of the condition likely increase PwCM’s need for dialogue with healthcare professionals and access to quality health information. For example, patient presentation has a diverse spectrum, with symptomatology ranging from numbness or dexterity issues indicative of mild dysfunction to quadriparesis and incontinence indicative of severe dysfunction [37]. DCM can be treated via a range of surgeries that address the degenerative changes on the cervical spine [38]. The decision to offer surgery and when to offer surgery is, however, nuanced, owing to risks and the potential for DCM to remain mild [39]. Further, surgery seldom leads to a full recovery. Instead, a varied but lifelong disability is expected [40, 41], including poor quality of life [32, 42], high unemployment [35, 41] and mental health difficulties [40]. The implications of this can involve can involve numerous different complex care pathways, consisting of distinct professional disciplines less familiar with DCM [43] necessitating that PwCM advocate for themselves. Estimated to affect 1 in 50 adults [31, 44], DCM is associated with significant healthcare resource utilisation, disability payments and lost productivity [45]. Given the rise in the aging population, intervention to help optimise patients’ long-term outcomes are critically needed [46].

Despite its prevalence, the information needs of PwCM have not been investigated to date. Notably, a recent scoping review of DCM educational resources found that the overwhelming majority (86%) are geared towards medical professionals [36, 47, 48]. Of these, more than 60% focused on surgical management, employing a highly specialised, technical language [36, 47, 48]. As increasingly most people turn to the internet to understand their condition and inform their treatment decisions [11, 49, 50], these findings illustrate an information-accessibility gap. Further, people with unresolved illness often prefer receiving information from clinicians [51] and healthcare professionals are best placed to deliver health information owing to their medical expertise and proximity to the patient’s specific circumstances [52]. An increased understanding of the information exchanged during healthcare consultations and PwCM’s informational needs is, therefore, necessary. Correspondingly, the present study used thematic analysis in order to determine what information is provided to PwCM during clinical interactions and to understand the nature of PwCM’s information needs. To achieve this understanding, the present study focused on the following two research questions:

1. What information is provided to PwCM during clinical interactions?
2. What, if any, information needs do PwCM have?

## Materials and methods

This qualitative study is reported in accordance with the Consolidated Criteria for Reporting Qualitative Research (COREQ) [53].

### Information needs

We employed Clarke and colleagues’ [22] conceptualisation of the term ‘information needs’ as information that participants expressed a desire for verbally or obtained by engaging in active information-seeking behaviour. We expanded this definition to include information that participants assigned utility to as this could represent an information need for others not in receipt of the same informational content.

### Participants

A convenience sampling strategy was employed [54]. Recruitment was advertised electronically within the Myelopathy.org Support Group, an online peer-to-peer support group hosted on Facebook [Meta, California, USA], between June 2021 and January 2022. Participants were eligible to participate if they were over 18 years of age, diagnosed with DCM and English speakers. Respondents were contacted by email and provided with written information on the purpose of the study, confidentiality, anonymity, and the voluntary nature of participating. No incentives were offered.

### Data collection

#### Materials

The topic guide (S1 Appendix) was comprehensively developed based on existing literature and using the expertise of our multidisciplinary research team. A draft schedule was first developed using patient booklets and information sheets [55–57], and collected information on the clinical evaluation, treatment, and care processes in DCM [31, 39, 58–61]. BMD, an Academic Neurosurgeon experienced in managing DCM, and PA, a Health and Care Professions Council (HCPC) Clinical Psychologist and experienced qualitative researcher, provided expert clinician opinion and the interview scheduled was further refined using their feedback. To ensure patients’ perspectives are integrated in the development of the topic guide, we referred to previous qualitative interviews with PwCM [62] and sought the perspective of a PwCM patient representative who did not participate in the study. Topics were structured chronologically. This approach has been employed by others investigating the information needs of people with a different degenerative condition [63]. Given the importance of adequate and sufficient information transfer from clinicians to patients in the context of chronic illness [64], questions explored the nature of information exchanged while prompts probed for potential information needs. The topic guide was piloted with a PwCM with medical background who confirmed its suitability.

#### Procedure

Ethical approval was obtained from Myelopathy.org (DCM Charity Number 1178673, reference DCM COINS-001). Semi-structured interviews were undertaken by the first author (IS), a postgraduate female Psychology student with previous theoretical and practical knowledge of conducting qualitative research. Interviews were held using Zoom, a videoconferencing app (Zoom Video Communications Inc., 2016). Data quality is not diminished by interviewing remotely [65]. Participants had no prior knowledge of IS. Before commencing, IS reiterated information sent in writing, emphasising that participation was entirely voluntary and that participants were free to take comfort breaks and end interviews at any time. Confidentiality and anonymity were explained and reassured. No repeat interviews were carried out. Field notes were taken during all interviews. IS liaised with participants via e-mail to arrange interviews and, therefore, had access to their names and contact details. E-mail messages were deleted once data were collected. To protect confidentiality, all participants’ names have been pseudo anonymised in this study.

#### Data analysis

Audio recorded interviews were transcribed verbatim and analysed according to Braun and Clarke’s [66] six-phase approach to thematic analysis. This method is designed to search for common or shared meanings [67] and is particularly suitable for exploratory work in understudied areas [68]. Given the lack of research on this topic within DCM, the codes were generated using an inductive coding process, a data driven method for coding that does not involve a predetermined theory or coding framework, and which prioritises participants’ meanings [66]. The coding process was semantic to ensure that codes were aligned with participants’ own explicit descriptions and the same unit of text could be assigned to more than one code. The first study author (IS) familiarised herself with the data by reading the transcripts in full several times, carefully and analytically. Coding was performed line by line, which allowed the data to be organised into meaningful categories [69]. IS started by categorising statements as ‘information received’ and ‘information needs’ and noted preliminary ideas for coding. During subsequent returns to the data, statements from participants’ transcripts within the two categories were assigned descriptive codes in accordance with their content. Data were analysed manually using Microsoft Word and Microsoft Excel. Several researchers have shown that standard office programs can be used to analyse qualitative data [70–73]. Briefly, interviews transcribed using Microsoft Word were transferred into Microsoft Excel, with each quote beginning with either “I” for the interviewer or “R” for respondents. Each row containing participants’ responses was assigned a code in the corresponding adjacent cell. When one quote contained information suited to more than one code, the row was copied underneath the corresponding quote and given a separate, additional code. The list of codes was kept in a separate sheet. As the coding list developed, returns to the first interviews were made to make adjustments to the initial codes. Throughout the process of coding, the first author spoke with the other authors, a HCPC Clinical Psychologist, an Academic Neurosurgeon specialised in DCM, and a Clinician Scientist. Codes were refined with subsequent readings of the transcripts based on patterns within the data and using feedback from the other three authors. Data were read and re-read to validate the integrity of the codes and to ensure that codes have explicit boundaries, are not interchangeable or redundant. During the refinement stage, some of the existing codes were adjusted or expanded to incorporate new information as necessary. When coding was complete, participant transcripts were combined in the same sheet and sorted by the codes assigned. This allowed for the identification of overlapping points and categorisation into thematic areas. Subthemes and main themes were derived using an inductive, ‘bottom-up’ approach [74] by inspecting the list of codes and associated quotes for common patterns. The inductive thematic analysis resulted in 61 codes which were subsequently grouped across three key main themes. Disagreements were resolved during project meetings and alternative solutions were discussed. Our team was able to pull from the vantage point of a PwCM Patient Representative who was not involved in the conduct of the study who verified that the codes, subthemes and main themes were representative of participants’ accounts. Employing a multidisciplinary approach was important to ensure a comprehensive interpretation of the data from differing perspectives [70]. All authors agreed the final thematic structure and content. Participants were sampled until data saturation was reached such that no new information was identified with respect to the research questions [75]. Verbatim quotes were abridged to exclude utterances (“uhm”), discourse markers (“like”, “you know”) or repetitions (“it, it”) considered immaterial to the communication itself. Square brackets with ellipses […] denote portions of omitted text.

## Results

### Participants

Twenty-eight respondents expressed an interest in participating in the study, eight of which remained uncontactable for unknown reasons. Detail of the participants who took part in in depth interviews are provided in Table 1 and S1 Table. Of the twenty who consented, thirteen were female (65%) and seven were male (35%). Ages ranged from 39 to 74 years of age, with a mean (SD) age of 58.75 (± 9.28). The majority (70%) had undergone an anterior cervical discectomy and fusion, while the remaining underwent posterior decompression (15%), a corpectomy (5%) or had not received surgical treatment at the time of the interview (10%). Nine participants (45%) were diagnosed in the past 0 to 2 years, seven (35%) within the past 3 to 10 years, and one (20%) within the past 12 years. Most participants (75%) were drawn from the UK. Interview lengths ranged between 52 minutes to 2 hours long, with an average of 1 hour and 25 min.

**Table 1:** Characteristics of interview participants.

Characteristic		Frequency n (%)
Gender	Female	13 (65%)
	Male	7 (35%)
Age Range	31–40	1 (5%)
	41–50	2 (10%)
	51–60	9 (45%)
	61–70	6 (30%)
	71–80	2 (10%)
Country	Canada	2 (10%)
	United Kingdom	15 (75%)
	United States	3 (15%)
Employment Status	Employed	8 (40%)
	Unemployed*	5 (25%)
	Retired	7 (35%)
Years Since Diagnosis	0–2 years	12 (60%)
	3–10 years	7 (35%)
	10+	1 (5%)
DCM Surgery	Yes	18 (90%)
	No	2 (10%)
Treatment	ACDF	14 (70%)
	Laminectomy	3 (15%)
	Corpectomy	1 (10%)
	Nonoperative Management	2 (10%)

*Unable to work due to disability

ACDF: anterior cervical discectomy and fusion

### Themes

Following thematic analysis, three main themes were identified as shown in Table 2 and S2 Table. The first theme, ‘Variation in the provision of information to PwCM during clinical interactions’ describes inconsistencies in the provision of information to PwCM during clinical interactions and the damaging implications of inadequate knowledge transfer. The second theme, ‘Variations in the information needs of PwCM’, reflects similarities and heterogeneity in participants’ information needs. Finally, the third theme, ‘Information that PwCM find useful’ details the type of information participants valued, alongside their perspective of its perceived utility.

**Table 2:** Main themes and subthemes.

Main Themes	Subthemes
**1. Variation in the provision of information to PwCM during clinical interactions**	1.1. Pathophysiology, symptomatology, clinical course and impact on quality of life The surgical treatment
**2. Variations in the information needs of PwCM**	2.1. The negative implications of lacking awareness of DCM 2.2. Pathophysiology, symptomatology, clinical course, impact on quality of life 2.3. The surgical treatment 2.4. Adjusting to life with disability
**3. Information that PwCM find useful**	3.1. The MRI as a pedagogic tool 3.2. The Support Group as a source of information 3.3. Other information that PwCM find useful

#### 1. Variations in the provision of information to PwCM during clinical interactions

*1*.*1*. *Pathophysiology*, *symptomatology*, *severity and clinical course*. The majority of participants were informed about their diagnosis prior to surgical treatment, either in writing or verbally. This information was considered fundamental, a necessary starting point closely linked to participant’s ability to research and understand the condition, and ask relevant questions during follow-up clinical interactions.

All three participants who had not received their diagnosis prior to surgical intervention reported wanting to have had this beforehand. The lack of communication about diagnosis had significant deleterious implications for one interviewee:

*“I was diagnosed in the doctor’s records in 2015*, *but they didn’t say anything to me*. *[…] I probably wouldn’t get my gait back and I would probably have myelopathy from the damage*.*”* (Claire)

Most participants reported receiving information concerning the cause of their symptoms which they understood to be spinal cord compression. This aspect of information about the condition’s pathophysiology appeared to be consistently well-conveyed by clinicians to patients during clinical interactions. However, some reported receiving little to no basic information regarding the permanent nature of damage incurred. This appeared to elicit an unwanted element of surprise, confusion and disappointment among those who assumed that surgical treatment would reverse the damage:

*“I had no idea until the date of the surgery when a nurse said to me*: *‘you can’t expect this to undo what you’ve got*, *it’ll keep it from getting worse’*. *And that’s the first indication I had of ‘oh man*, *they’re not going to fix me completely’*. *That’s the first time it was mentioned to me*.*”* (Nick)

*1*.*2*. *The surgical treatment*. The majority of participants were informed about the aim of surgery and understood this to be decompressing the spine in order to halt the progression of the illness. For some, detailed information on this topic was regarded as a means of appraising whether their doctor was acting in line with best practice and ensuring non-surgical treatment avenues had been explored before operative management was offered:

“*It made me appreciate he wasn’t just jumping on the route of ‘you need surgery’*. *Because I googled so much and saw so many people having physiotherapy and steroids*, *I was still confused about why I needed the final part of it*, *the worst part of it*. *He explained why*: *that there was nothing else that would alleviate the compression other than removing the bone spurs that were against it*.” (Mary)

All interviewees reported receiving information concerning surgical risks and complications, although the nature of information received on this topic varied ranging from infection, blood clots, stroke, cerebrospinal fluid leak into the spinal cord, permanent damage leading to paralysis, damage to the vocal cords, to death. Information was received verbally as part of the clinical consultation and in writing, as part of the process of collecting informed consent prior to surgery. Among those who considered this information useful, some attributed its utility to being able to plan ahead, set expectations for care, and ensure they are able to “make an informed decision” (Emma) before consenting to an invasive surgical procedure. Notably, one participant remarked on the importance of the timing of delivery of information: *“On the morning of the surgery the anaesthetist came and said I need to tell you because this would be a very long surgery and you’ll be with your head down*, *this could put pressure on your optic nerve and there’s some risk of going blind*. *That was not good to hear right before going to the operating room*. *[…] It was very distressing so I remembered it*, *my husband also said–wow that was really stupid to tell you that right before surgery*.” (Karen)

The amount and nature of preoperative information participants received varied significantly. A dichotomy in the provision of information was noted. For example, some reported receiving no preoperative guidance: “*He didn’t say what to do*, *there was nothing… He just put me on his waiting list”* (Tim). Others assessed that they had received comprehensive informational support that adequately prepared for the operative process: *“He explained what was going to happen and everything else*, *what was going to happen during surgery and post-surgery*. *I was quite well geared up for it when the day happened”* (Jay). Although most interviewees reported receiving information about the duration of the postoperative neurological recovery, this appeared to vary significantly, with participants reporting recovery durations of 6, 9, 12 and 24 months.

#### 2. Variations in the information needs of PwCM

*2*.*1*. *The negative implications of lacking awareness of DCM*. None of the interviewees had any awareness of DCM prior to being diagnosed with the condition. There was a clear drive among participants to determine the origin of their symptoms, for which three approaches were employed. Many spoke about turning to internet-based research, anecdotally referred to as ‘Dr Google’ (Sara) or ‘Professor Google’ (Jo), in an attempt to diagnose themselves: *“I just done a lot of research myself*, *on the internet*, *and come to the conclusion that it was fibromyalgia*.*”* (Emma)

However, unawareness of the condition impeded participants’ ability to find relevant information. The diversity of symptoms and lacking the name of the disease were noted by some as the main roadblocks to conducting efficient internet searches: *“Had I known the term myelopathy and knew this could happen maybe I would type that in and then got a whole host of my symptoms*, *but putting your symptoms in doesn’t seem to work”* (Lynn). For others, the primary strategy for obtaining information involved consulting their primary care physician on the basis of the view that ‘googling’ one’s symptoms was “more confusing than helpful” (Paul) or would elicit “all sorts of bum information’ (Tim). Some engaged in both strategies. Unawareness of the condition, however, had significant implications for participants, particularly for those unable to obtain a diagnosis for years. Many attributed their symptoms to other conditions such as fibromyalgia, multiple sclerosis, menopause, arthritis, carpel tunnel, lower back problems or amyloidosis: “*My mom is a multiple-sclerosis patient*, *so I thought I was the same*” (Paul).

A trend of diagnostic delays resulting in preventable deterioration was identified. Participants spoke about the importance of being diagnosed early and having awareness of the condition as an essential: “*Time is of the essence in terms of spinal cord compression and getting it decompressed*. *I feel I lost some time there as I was deteriorating*” (Karen). Some spoke about personal safety issues associated with lacking awareness of DCM including developing suicidal thoughts due to intolerable pain or engaging in physical activity which would otherwise be contraindicated when the spinal cord is compressed: *“One [doctor] even told me to go for a run*, *which is really quite dangerous now in my situation”* (Emma).

*“I actually did consider in my mind*: *‘I don’t want to be here anymore*, *with this pain*, *if nobody’s going to help me and I’m going to be in this pain forever*, *I don’t want to be here anymore’*. *And I had that thought and I had a plan in my mind*. *How to not be here anymore*. *Luckily*, *I didn’t act on it”* (Lynn)

*2*.*2*. *Pathophysiology*, *symptomatology*, *severity and clinical course*. Participants reported broad knowledge gaps with respect to the disease mechanisms that lead to DCM, its severity, symptomatology and prognosis. Interviewees valued information about the disease process, although their preference for information regarding the condition’s pathophysiology, symptomology, severity and clinical course appeared to vary. Several expressed a desire for general information concerning the condition:

*“It would have been quite nice to have something like a fact sheet about the condition*, *even if it’s just a basic thing*.*”* (Elaine)

Others reported more specific knowledge deficits that they would have liked addressed, with prognosis being a key area of interest for some. The desire for information regarding the typical clinical course associated with DCM appeared to be related to both managing expectations and being able to plan in advance. While some found the idea of never making a full or very limited recovery “incredibly depressing” (Jo), others valued having “clarity over a progressive disease” (Mandy). Some considered realistic prognostic information an important means of extending clarity over circumstances to family members: “*Although the consultant is talking to me*, *I’m part of a family and other people want to understand this [the prognosis]*. *This will impact on my partner*, *my wife and her plans*” (Robert).

Notably, some participants emphasised the need for personalised communications regarding one’s clinical course by expressing that their desire for prognostic information was contingent on its favourableness: *“If 80% of people make a full recovery*, *I’d feel more hopeful*, *rather than if they said only 40% do*. *So*, *it depends on what statistics they were giving me*, *really”* (Lynn).

Others reported wanting to have had knowledge about the permanent nature of damage caused by the condition. These participants were among those who had not been made aware of the irreparable nature associated with spinal cord injury due to DCM.

*“I would have liked to know that my spinal cord was permanently damaged*, *that it was not gonna get any better*.*”* (Claire)

There was a sense of confusion among participants with respect to the clinical manifestation of DCM, “*the whole constellation of symptoms it can cause*” (Angela), which was expressed as questions about existing, novel and potential future symptoms: “Should I be experiencing this, is it normal to feel like this?” (Tim). There was an overarching expectation that this information should be offered by the healthcare professionals as part of the clinical consultation or in the form of auxiliary materials: “*I’m having new symptoms*, *new pain and why is this*? *I don’t know why… I tried googling*, *I can’t find*, *is this common*, *normal*? *I could maybe get worse*, *my pain*, *I thought I’d be getting better not worse*. *Information like that on future symptoms and if I encounter these new issues is it related or not*?” (John). Drivers for this information need appeared to include the desire to exclude other illnesses, adequate use of financial resources, and managing personal safety risks and the unpredictability and impact of DCM on quality of life: *“If you know it is related to myelopathy*, *you can just put a label on that and say that’s from that*. *But if I were to develop something else and then it’s not related to myelopathy then I need to ask for a bit more help from somewhere else*.*”* (Lynn)

*“Just to put 2 and 2 together*, *with the sensations I have*. *[…] I can help prevent falls for myself and that kind of thing too*.*”* (Angela)

Reflecting on the magnitude of impact DCM exerted on his quality of life, one participant stated a desire for information regarding the severity of DCM: *“If they had a scale on myelopathy 1–5 or 1–10*, *in their best opinion you’re in the scale on number 3*. *Any advice that relates to number 3 you could see what you could expect and what you might not expect*. *I can’t stress enough*: *if it’s quite bad*, *it just screws your life*. *And to be prepared for that”* (George).

*2*.*3*. *The surgical treatment*. Most participants described a lack of informational support during the perioperative environment that seemed rooted in communication gaps or ineffective communication of information leading to fragmented care. This was recognised retrospectively through their own research and as a result of having gone through the process. There was sense of feeling challenged and alone during the process of navigating complex care situations, with variations in the nature of information desired. For example, some were primarily concerned with ensuring physical safety: *One of the things I should have been told is not to drive […] I’d get spasms in my body […] if you had a spasm while driving*, *you could cause an accident*, *so I should have been told not to drive”* (Lynn). Others were interested in practical, logistical details associated with the hospital stay: *“How long I’m going to be in there*? *Visiting*? *I’ve also got a gluten allergy so what happens with food*? *Do I need to take money*? *Just general*, *what happens with my stuff when I’m in surgery*? *Where does that go*?*”* (Emma). One participant was of the view that the topic of prehabilitation is warranted in preoperative clinical conversations: *“I think it would be helpful to discuss prehab just because it’s something the person could do that gives them a bit of agency*, *rather than just sitting and waiting until the surgery is scheduled”* (Karen).

Others reported needing information regarding the surgical methods, risks and complications such as the development of adjacent segment disease, swallowing difficulties, and risk of paralysis.

Postoperative informational support gaps were also described by some interviewees. In particular, being unequipped with sufficient knowledge during the recovery period appeared to augment participants’ confusion, anxiety and even fostered a lack of trust in health care.

For these participants, the lack of relevant information fostered a sense of being unable to engage in forward planning and adequately prepare themselves physically, mentally, socially and practically to influence recovery in the best possible way or to engage in adequate long-term management of the condition. *“I still don’t know what symptoms I’m supposed to be looking for*. *[…] I’d googled that as well*, *because I did have a problem with my wound”*, one participant (Mary) echoed thinking about the lack of postoperative guidance received. “I just wasn’t given any information so I’m not sure what I would tell someone else”, she continued. Reflecting on his desire to engage in social activities with his family, another echoed: *“Another thing*, *very important*, *which I asked a lot was […] if I’d done x*, *y*, *z and it’s painful*, *so what*? *Am I going to cause any further damage*? *That’s incredibly important to be told*.*”* (George)

Concerning the surgical approach, some participants were of the view that one “*goes with what the surgeon suggests because they’re the expert*” (Lucy). The rationale behind this perspective was well illustrated by one participant: *“I had no input at all*. *I mean*, *and why should I*? *Because I am not the expert*. *I go to the expert*. *He tells me what he will offer and what he offered was what I had*.*”* (Jo). Conversely, others voiced a desire for “*a little bit more information*” (Sara, line 161) and higher degree of involvement in the decision-making process: *“I didn’t know the pros and cons of which surgery*, *why some people get this one and not the other*, *why did they recommend this option for me*, *is it better or not better*? *I still don’t know*. *[…] I’m not sure it was the right decision”* (John).

Lastly, some reported needing information concerning medications, relevant both preoperatively and postoperatively. The main problem encountered by most participants in this regard, appeared to be a general lack of guidance on this topic: *“I found out by googling it*. *[…] That you weren’t meant to take them [NSAIDs] because it possibly stopped fusion*. *I didn’t get told that by anybody*. *[*…*] They should tell you if you’re not meant to be taking them because it could be a big deal”* (Mary).

*“Baclofen for muscle spasticity*, *that was a game changer for me because*, *because nobody had mentioned that at that point*. *[…] People need to know that there are medications available to help counteract that*. *Because someone’s left permanently damaged*, *spinal cord wise*, *that’s why that didn’t resolve after surgery”* (Lucy)

*2*.*4*. *Adjusting to life with a disability*. Participants described a lack of postoperative care coordination, which made adjusting to life with a potential new disability challenging and difficult to navigate. Those who were not retired or about to retire at the time of diagnosis or treatment shared uncertainties concerning their ability to return to work: *“I’ve been job hunting through this whole thing too and my doctors all know that*, *but there’s no advice […] I’m not told*, *I know nothing”* (Angela).

*“I don’t know how much time to take off from work*, *it says on the website 4–12 weeks*, *I feel like I’m left making decisions by myself*.*”* (Elaine)

Participants emphasised the importance of being signposted to avenues of information regarding benefits and financial support: One woman echoed this line of thought: *“All I got in terms of employment advice was no heavy lifting*. *That was it*. *[…] Just signpost people to the help that they need to make those decisions about whether they need to take disability benefits or not*.*”* (Lucy)

Some participants also expressed a need for information regarding costs associated with assistive equipment or “*adaptations in the home”* (Robert): “*There’s just a lot of financial implications as to what you may need to*, *you know*, *what do you need to work out*, *what do you need to build your strength back up*, *what kind of gadgets do you need at home as far as weights or maybe one of those bicycles you can do from a chair for your own rehabilitation”* (Claire).

Information concerning the impact of DCM on a person’s mental health was considered key by most participants. Many shared that they had struggled accepting their diagnosis, its impact on their quality of life, and that processing the long-term clinical course associated with the condition was a fundamental challenge. There was a sense that mental health guidance was an instrumental component of care and should become common practice given the psychophysiological strains associated with the condition: *“I think there should be support available or even if it’s phone numbers that people can contact*. *I struggle with the fact that I might not be better*, *or I’m stuck with these symptoms*.*”* (Mandy)

#### 3. Information that PwCM find useful

*3*.*1*. *The MRI scan as a pedagogic tool*. Participants who were shown and explained their MRI scan reported an enhanced understanding of the condition, the need for and urgency of treatment, as well as a sense of experiential validation. Essentially, the MRI scan provided “*clear*, *visual evidence of what the problem was”* (Tim, line 128). As summarised by one participant, “*a picture is worth a thousand words*” (Nick, line 77).

Conversely, those who did not have the opportunity to see their MRI spoke about wanting to have had a visual representation of the condition: *“You see physiotherapists in offices they have a mock-up spine*, *don’t they*, *of plastic*? *If they’d showed me that and said ‘look*, *this is what’s happening to your spine and because of that that’s what is causing your gait problems*, *bowel and bladder problems’*. *I’d just found it a lot easier to cope with*.*” (Marion)*

*3*.*2*. *The support group as a source of information*. Interviewees described using the Myelopathy.org Facebook community as a means of supplementing information received or addressing information deficits: “*They have 2000 people*, *so if you ask a question guaranteed someone has experienced it”* (Paul). The nature of the information content sought was wide-ranging, extending from information about the condition, medications, pre- and post-operative guidance, assistive equipment, symptoms, finance and employment, to impact on mental health: *“Most of the information I got about the surgery and what to expect has come from the Support Group of those that have already had it*.*”* (Emma)

The informational support provided by the community appeared to also ameliorate varying levels of distress experienced by participants associated with lacking information on a given topic: *“I was so worried about the trembling and the worms*, *I posted about that and I got some very sensible replies*, *they said it’s quite normal*. *Most people like me*, *nobody seems to bother to tell you why*. *But apparently*, *it’s normal when you’ve got myelopathy to get these*.*”* (John)

Although the support group appeared to be valued as an information resource, some cautioned against being exposed to disquieting information posted by other members: *“People were degenerative after surgery*, *people were in massive pain years later*, *they’d had to go in and have it done again*, *they weren’t on the right pain relief […] So*, *I stopped reading it*.*”* (Tim)

*3*.*3*. *Other information that PwCM find useful*. The spectrum of knowledge that interviewees found useful was broad, ranging from information about the condition, its severity and treatment to the long-term clinical course. The perceived utility of information appeared to parallel participants’ individual preferences for information quantity, their understanding of their circumstances and expectations. For example, some found understanding the pathophysiology of the condition useful towards their emotional health and in assisting their understanding of the need for surgery: *“I blamed myself*. *I thought I’ve tripped*, *I’ve failed*, *I’ve lifted something*. *I felt like I caused it to myself*. *[…] Learning the pathology and origins of it would be helpful to not blame yourself*. *Because you’re going through so much anyway”* (Lynn). For others, the provision of information supported their physical safety during the postoperative recovery period: “*It stopped me doing things I may have done if I hadn’t asked them*. *I would have probably gone on and done anything really*, *I think that would have damaged me”* (Mary).

Knowledge of condition severity assisted one participant understand the need for surgery: *“If your symptoms were mild*, *you didn’t need surgery*. *But if they were moderate or severe then you had to have surgery and I could see the words moderate and severe there somewhere*, *so that also led me to believe the surgeon was correct*.*”* (Mary).

Participants also valued information concerning the neurological recovery as a means of managing expectations and assessing their likely long-term clinical course: *“If you were told no timescale at all*, *your whole life would just be hoping and hoping to get better*. *[*…*] I think people want a timeline*. *Everyone I’ve spoken to has wanted a timeline*.*” (Lynn)*

## Discussion

This study identified that the provision of information to PwCM during clinical interactions varies, sometimes carrying significant long-term implications for an individual’s health. Prominent areas of informational support gaps included the condition, its treatment, the subsequent recovery, and adjusting to life with residual disability. The use of patients’ MRI scans as an educational tool by healthcare professionals highlighted the benefits of improved education using minimal effective guidance and already existing resources. Overall, these results indicate that informational support in DCM warrants improvement.

Our findings demonstrate how medical information that is poorly explained or omitted altogether can have life-altering implications [76]. Providing patients with information about the condition, starting with the correct nomenclature is crucial, particularly for a condition that has been historically known by over 11 different names such as DCM [77]. As illustrated by the one participant, adequate communication of diagnosis is key to avoiding progressive neurological dysfunction that is irreversible [31, 78].

Beyond awareness of the diagnosis, many other information gaps were identified which suggests that PwCM participants possessed inadequate levels of knowledge about their condition. This was illustrated by interviewees’ hunger for particulars about the disease pathophysiology, symptomatology, clinical course and surgical management of DCM and concurs with current academic literature suggesting that inadequate information transfer is a frequently encountered issue in clinical practice [79–86]. Downstream of inconsistent information provision, PwCM struggled with engaging in self-management strategies. Again, prior research has identified inappropriate information acquisition and management as a significant barrier to engaging in adequate self-management strategies, a key skill for individuals with lifelong conditions [87]. Our results show that PwCM were interested in health information and were, for the most part, engaging in active information seeking strategies to better understand the condition. Indeed, a general understanding of one’s illness is requisite for participation in and adherence to treatment [88–92]. The information sought by participants in respect to disease mechanisms was general and nonspecific, but this is to be expected as health information seeking behaviour amplifies over time as patients engage more intensively with an expanding understanding of their condition [51, 93].

Knowledge of one’s likely clinical course can inform life-altering care and treatment decisions [94–97]. That participants in this study appreciated or wanted to enhance their knowledge of the clinical course highlights the importance of prognosticating in the context of life-limiting disorders as a major pillar supporting patient-centred care [96, 98–100]. Despite this, we found that conversations on this topic were of poor quality or omitted altogether which is congruent with several lines of evidence on this topic [101–103]. This information should, therefore, be made available to PwCM as its provision has important implications for a chronic illness patient’s onward health decision making, mental and emotional health and openness to treatment [104, 105]. However, the results suggest that care should be taken in the manner of its delivery. Indeed, others have found that patients’ preferences for the level of detail concerning prognosis can vary significantly [106–108], emphasising that its communication should be tailored to the patient [79, 108–113]. This is in line with good practice recommendations encouraging clinicians to detach themselves from paternalistic medical expert roles and take a tailored approach to imparting biomedical knowledge and uncertainty in a manner that is personalised to the patient and their best interests [114].

Similarly, participants’ desire for symptomatology information reflected a common need of chronic illness sufferers to appraise their current health status by relating it to the current manifestation of the illness [115]. Aside from being an important component of health-related quality of life [116], symptom status in DCM can be prodromal of or denote degeneration of untreated levels of the cervical spine [31, 117]. Increasing patients’ awareness of DCM symptoms therefore has important clinical implications for their physical safety.

We also identified a lack of comprehensive perioperative informational support [18, 118, 119], illustrated by PwCM participants’ numerous and varied questions pertaining to this critical moment of care. That interviewees’ appraisal of the level of perioperative quality of care appeared to parallel the perceived success of information transfer highlights the importance of quality communication during this critical phase of care [120]. Poor perioperative informational support appeared to interfere with various aspects of participants’ safety during recovery, ability to engage in self-management strategies [121] and, for some, appeared to act as a source of anxiety and confusion. Uninformed participants also carried a higher risk of postoperative safety challenges [122].

On the other hand, the pedagogic use of participants’ MRI scan, which plays a central role throughout the management of DCM [123], by healthcare professionals illustrates how simple, resource-light interventions can improve outcomes in DCM. Our findings were in line with research showing that diagnostic imaging can enhance patients’ understanding of their condition and validate their lived experience [124, 125].

Participants unanimously agreed that information concerning the mental health and financial implications of living with DCM should be provided as part of the care package. Most incurred a level of financial burden as a result of living with DCM-induced disability [40]. Of those still working at the time this study was undertaken, many questioned their ability to return to work, a common source of concern among PwCM [35]. Anxiety and depression are common DCM-induced functional deficit [31, 126] and PwCM can experience suicidal ideation as a result of living with the condition [40]. These challenges are likely magnified by the poor awareness of DCM [127], again referenced in this study. Joined-up healthcare strategies addressing these information and support gaps are therefore pressingly needed to ensure patient safety.

Whilst the finding that PwCM found the online support group to be a useful information resource may reflect the nature of the study sampling, chronic illness populations acquire information from both professional and lay sources [128–131]. That online communities may have a didactic benefit whereby participants can address knowledge deficits has been previously documented [132–135]. Signposting PwCM to relevant online communities may therefore represent a complementary cost-effective avenue for improving patient education.

### Limitations

Prompting participants’ recollection of information post-event carries a risk of recall bias, which could be overcome by a longitudinal pre-, during- and post-event approach [136]. The involvement of potential lower socioeconomic or elderly participants was likely limited by the eligibility requirements including access to an internet connected device. Lastly, recruiting participants from Myelopathy.org community may have overrepresented the impact of an online support group as a useful information resource.

## Conclusion

The current informational gap described by PwCM is antithetic with the concept of person-centred care [92, 137] and the notion of increased self-management as a solution to the strain on healthcare services in chronic illness [90–92]. Our findings show that PwCM need reliable, personalised informational support throughout the course of their clinical care. Efforts must now turn to developing solutions for the provision of a comprehensive, patient-centred information exchange in DCM. To address this challenge, we will develop the first DCM Diagnosis Core Information Sets (CISs), a collection of information items designed to aid professionals, patients and their carers ensure that key information is discussed with patients at diagnosis. CISs have been used by others to facilitate educational conversations prior to surgical intervention with the aim of collecting patients informed consent [138–141]. As healthcare resources are limited, being able to prioritise the most-needed information will make clinical encounters more meaningful and useful to patients [142]. Future lines of investigation should seek to develop informational support tools for use by PwCM at key points in their clinical care.

## Supporting information

S1 Appendix Interview schedule. (DOCX)

S1 Table Characteristic of interview participants. (DOCX)

S2 Table Main themes and subthemes. (DOCX)

## References

[1] Husson O, Thong MS, Mols F, Smilde TJ, Creemers GJ, van de Poll-Franse LV. Information provision and patient reported outcomes in patients with metastasized colorectal cancer: results from the PROFILES registry. J Palliat Med. 2013;16(3):281–8. doi: 10.1089/jpm.2012.0430 23437833 PMC3583247

[2] Husson O, Mols F, van de Poll-Franse LV. The relation between information provision and health-related quality of life, anxiety and depression among cancer survivors: a systematic review. Ann Oncol. 2011;22(4):761–72. doi: 10.1093/annonc/mdq413 20870912 PMC3065875

[3] Gillani SM, Nevill A, Singh BM. Provision of structured diabetes information encourages activation amongst people with diabetes as measured by diabetes care process attainment: the WICKED Project. Diabet Med. 2015;32(7):865–71. doi: 10.1111/dme.12737 25764229

[4] Grobosch S, Kuske S, Linnenkamp U, Ernstmann N, Stephan A, Genz J, et al. What information needs do people with recently diagnosed diabetes mellitus have and what are the associated factors? A cross-sectional study in Germany. BMJ Open. 2018;8(10):e017895. doi: 10.1136/bmjopen-2017-017895 30385437 PMC6252653

[5] Rodger S, Bench S. Education provision for patients following a spinal cord injury. Brit J Nursing. 2019;28(6):377–81. doi: 10.12968/bjon.2019.28.6.377 30925253

[6] Christalle E, Zill JM, Frerichs W, Härter M, Nestoriuc Y, Dirmaier J, et al. Assessment of patient information needs: A systematic review of measures. PLoS One. 2019;14(1):e0209165. doi: 10.1371/journal.pone.0209165 30703103 PMC6354974

[7] Lubkin IM LP. Chronic Illness: Impact and Intervention. 8th ed: Jones & Bartlett Learning; 2013.

[8] Wagner EH, Austin BT, Von Korff M. Organizing care for patients with chronic illness. Milbank Q. 1996;74(4):511–44. 8941260

[9] Xiong T, Turner RM, Wei Y, Neal DE, Lyratzopoulos G, Higgins JP. Comparative efficacy and safety of treatments for localised prostate cancer: an application of network meta-analysis. BMJ Open. 2014;4(5):e004285. doi: 10.1136/bmjopen-2013-004285 24833678 PMC4024605

[10] Lorig KR, Sobel DS, Ritter PL, Laurent D, Hobbs M. Effect of a self-management program on patients with chronic disease. Eff Clin Pract. 2001;4(6):256–62. 11769298

[11] Scherer LD, Matlock DD, Allen LA, Knoepke CE, McIlvennan CK, Fitzgerald MD, et al. Patient Roadmaps for Chronic Illness: Introducing a New Approach for Fostering Patient-Centered Care. MDM Policy Pract. 2021;6(1):23814683211019947. doi: 10.1177/23814683211019947 34277949 PMC8255605

[12] Coulter A, Entwistle VA, Eccles A, Ryan S, Shepperd S, Perera R. Personalised care planning for adults with chronic or long-term health conditions. Cochrane Database Syst Rev. 2015;2015(3):Cd010523. doi: 10.1002/14651858.CD010523.pub2 25733495 PMC6486144

[13] Watt S. Clinical decision-making in the context of chronic illness. Health Expect. 2000;3(1):6–16. doi: 10.1046/j.1369-6513.2000.00076.x 11281907 PMC5081080

[14] Tinetti ME, Naik AD, Dindo L, Costello DM, Esterson J, Geda M, et al. Association of Patient Priorities-Aligned Decision-Making With Patient Outcomes and Ambulatory Health Care Burden Among Older Adults With Multiple Chronic Conditions: A Nonrandomized Clinical Trial. JAMA Intern Med. 2019;179(12):1688–97. doi: 10.1001/jamainternmed.2019.4235 31589281 PMC6784811

[15] Lim CY, Berry ABL, Hirsch T, Hartzler AL, Wagner EH, Ludman EJ, et al. Understanding What Is Most Important to Individuals with Multiple Chronic Conditions: A Qualitative Study of Patients’ Perspectives. Journal of General Internal Medicine. 2017;32(12):1278–84. doi: 10.1007/s11606-017-4154-3 28849368 PMC5698221

[16] Peart A, Lewis V, Barton C, Brown T, White J, Gascard D, et al. Providing person-centred care for people with multiple chronic conditions: protocol for a qualitative study incorporating client and staff perspectives. BMJ Open. 2019;9(10):e030581. doi: 10.1136/bmjopen-2019-030581 31594885 PMC6797345

[17] Beisecker AE, Beisecker TD. Patient information-seeking behaviors when communicating with doctors. Med Care. 1990;28(1):19–28. doi: 10.1097/00005650-199001000-00004 2296214

[18] Keulers BJ, Scheltinga MR, Houterman S, Van Der Wilt GJ, Spauwen PH. Surgeons underestimate their patients’ desire for preoperative information. World J Surg. 2008;32(6):964–70. doi: 10.1007/s00268-008-9581-1 18408963 PMC2386849

[19] Waitzkin H. Information Giving in Medical Care. Journal of Health and Social Behavior. 1985;26(2):81–101. doi: 10.2307/2136599 4031436

[20] Kinnersley P, Edwards AGK, Hood K, Cadbury N, Ryan R, Prout H, et al. Interventions before consultations for helping patients address their information needs. Cochrane Database of Syst Rev. 2007(3):CD004565. doi: 10.1002/14651858.CD004565.pub2 17636767 PMC9036848

[21] Coulter A, Entwistle V, Gilbert D. Sharing decisions with patients: is the information good enough? BMJ. 1999;318(7179):318–22. doi: 10.1136/bmj.318.7179.318 9924064 PMC1114785

[22] Clarke MA, Moore JL, Steege LM, Koopman RJ, Belden JL, Canfield SM, et al. Health information needs, sources, and barriers of primary care patients to achieve patient-centered care: A literature review. Health Informatics J. 2016;22(4):992–1016. doi: 10.1177/1460458215602939 26377952

[23] Rees S, Williams A. Promoting and supporting self-management for adults living in the community with physical chronic illness: A systematic review of the effectiveness and meaningfulness of the patient-practitioner encounter. JBI Libr Syst Rev. 2009;7(13):492–582. doi: 10.11124/01938924-200907130-00001 27819974

[24] Schwappach DL, Mülders V, Simic D, Wilm S, Thürmann PA. Is less more? Patients’ preferences for drug information leaflets. Pharmacoepidemiol Drug Saf. 2011;20(9):987–95. doi: 10.1002/pds.2212 21796721

[25] Wong S, Walker JR, Carr R, Graff LA, Clara I, Promislow S, et al. The information needs and preferences of persons with longstanding inflammatory bowel disease. Can J Gastroenterol. 2012;26(8):525–31. doi: 10.1155/2012/735386 22891177 PMC3414474

[26] Matter B, Feinberg M, Schomer K, Harniss M, Brown P, Johnson K. Information needs of people with spinal cord injuries. J Spinal Cord Med. 2009;32(5):545–54. doi: 10.1080/10790268.2009.11754556 20025150 PMC2792460

[27] Lewis CL, Pignone MP. Promoting informed decision-making in a primary care practice by implementing decision aids. N C Med J. 2009;70(2):136–9. 19489371 PMC3213756

[28] Wagner EH. Chronic disease management: what will it take to improve care for chronic illness? Eff Clin Pract. 1998;1(1):2–4. 10345255

[29] Keij SM, van Duijn–Bakker N, Stiggelbout AM, Pieterse AH. What makes a patient ready for Shared Decision Making? A qualitative study. Patient Educ Couns. 2021 Mar. 104(3):571–7. doi: 10.1016/j.pec.2020.08.031 32962880

[30] Barnett G, Swart M. Shared decision making for high-risk surgery. BJA Educ. 2021 Aug;21(8):300–6. doi: 10.1016/j.bjae.2021.03.006 34306731 PMC8283422

[31] Davies BM, Mowforth OD, Smith EK, Kotter MR. Degenerative cervical myelopathy. BMJ. 2018 Feb 22;360:k186. doi: 10.1136/bmj.k186 29472200 PMC6074604

[32] Hirayama Y, Mowforth OD, Davies BM, Kotter MRN. Determinants of quality of life in degenerative cervical myelopathy: a systematic review. Br J Neurosurg. 2023 Feb;37(1):71–81. doi: 10.1080/02688697.2021.1999390 34791981

[33] Boerger TF, Davies BM, Sadler I, Sarewitz E, Kotter MRN. Patient, sufferer, victim, casualty or person with cervical myelopathy: let us decide our identifier. Integrated Healthcare Journal. 2020;2(1):e000023. doi: 10.1136/ihj-2019-000023 PMC10327448 37441311

[34] Khan O, Badhiwala JH, Grasso G, Fehlings MG. Use of Machine Learning and Artificial Intelligence to Drive Personalized Medicine Approaches for Spine Care. World Neurosurg. 2020 Aug;140:512–8. doi: 10.1016/j.wneu.2020.04.022 32797983

[35] Pope DH, Mowforth OD, Davies BM, Kotter MRN. Diagnostic Delays Lead to Greater Disability in Degenerative Cervical Myelopathy and Represent a Health Inequality. Spine (Phila Pa 1976). 2020 Mar 15;45(6):368–77. doi: 10.1097/BRS.0000000000003305 31658234

[36] Umeria R, Mowforth O, Grodzinski B, Karimi Z, Sadler I, Wood H et al. A scoping review of information provided within degenerative cervical myelopathy education resources: Towards enhancing shared decision making. PLoS One. 2022;17(5):e0268220. doi: 10.1371/journal.pone.0268220 35588126 PMC9119544

[37] Kalsi-Ryan S, Karadimas SK, Fehlings MG. Cervical spondylotic myelopathy: the clinical phenomenon and the current pathobiology of an increasingly prevalent and devastating disorder. Neuroscientist. 2013;19(4):409–21. doi: 10.1177/1073858412467377 23204243

[38] Kato S, Ganau M, Fehlings MG. Surgical decision-making in degenerative cervical myelopathy–Anterior versus posterior approach. J Clin Neurosci. 2018 Dec;58:7–12. doi: 10.1016/j.jocn.2018.08.046 30279123

[39] Milligan J, Ryan K, Fehlings M, Bauman C. Degenerative cervical myelopathy: Diagnosis and management in primary care. Can Fam Physician. 2019 Sep;65(9):619–24. 31515310 PMC6741789

[40] Khan DZ, Fitzpatrick SM, Hilton B, McNair AG, Sarewitz E, Davies BM, et al. Prevailing Outcome Themes Reported by People With Degenerative Cervical Myelopathy: Focus Group Study. JMIR Form Res. 2021 Feb;5(2):e18732. doi: 10.2196/18732 33533719 PMC7889422

[41] Fehlings MG, Ibrahim A, Tetreault L, Albanese V, Alvarado M, Arnold P, et al. A global perspective on the outcomes of surgical decompression in patients with cervical spondylotic myelopathy: results from the prospective multicenter AOSpine international study on 479 patients. Spine (Phila Pa 1976). 2015;40(17):1322–8. doi: 10.1097/BRS.0000000000000988 26020847

[42] Stoffman MR, Roberts MS, King JT, Jr. Cervical spondylotic myelopathy, depression, and anxiety: a cohort analysis of 89 patients. Neurosurgery. 2005;57(2):307–13; discussion -13. doi: 10.1227/01.neu.0000166664.19662.43 16094160

[43] Hilton B, Tempest-Mitchell J, Davies B, Kotter M. Route to diagnosis of degenerative cervical myelopathy in a UK healthcare system: a retrospective cohort study. BMJ Open. 2019;9(5):e027000. doi: 10.1136/bmjopen-2018-027000 31061045 PMC6501948

[44] Smith SS, Stewart ME, Davies BM, Kotter MRN. The Prevalence of Asymptomatic and Symptomatic Spinal Cord Compression on Magnetic Resonance Imaging: A Systematic Review and Meta-analysis. Global Spine J. 2021 May;11(4):597–607. doi: 10.1177/2192568220934496 32677521 PMC8119927

[45] Davies BM, Phillips R, Clarke D, Furlan JC, Demetriades AK, Milligan J, et al. Establishing the Socio-Economic Impact of Degenerative Cervical Myelopathy Is Fundamental to Improving Outcomes [AO Spine RECODE-DCM Research Priority Number 8]. Global Spine Journal. 2022;12(1_suppl):122S–9S. doi: 10.1177/21925682211039835 35174730 PMC8859704

[46] The Lancet Neurology. A focus on patient outcomes in cervical myelopathy. Lancet Neurol. 2019;18(7):615. doi: 10.1016/S1474-4422(19)30168-1 31202463

[47] Davies BM, McHugh M, Elgheriani A, Kolias AG, Tetreault LA, Hutchinson PJ, et al. Reported Outcome Measures in Degenerative Cervical Myelopathy: A Systematic Review. PLoS One. 2016;11(8):e0157263. doi: 10.1371/journal.pone.0157263 27482710 PMC4970758

[48] Davies BM, McHugh M, Elgheriani A, Kolias AG, Tetreault L, Hutchinson PJ, et al. The reporting of study and population characteristics in degenerative cervical myelopathy: A systematic review. PLoS One. 2017;12(3):e0172564. doi: 10.1371/journal.pone.0172564 28249017 PMC5332071

[49] Eysenbach G, Jadad AR. Evidence-based patient choice and consumer health informatics in the Internet age. J Med Internet Res. 2001 Apr-Jun;3(2):E19. doi: 10.2196/jmir.3.2.e19 11720961 PMC1761898

[50] Morahan-Martin JM. How internet users find, evaluate, and use online health information: a cross-cultural review. Cyberpsychol Behav. 2004 Oct;7(5):497–510. doi: 10.1089/cpb.2004.7.497 15667044

[51] Gille S, Griese L, Schaeffer D. Preferences and Experiences of People with Chronic Illness in Using Different Sources of Health Information: Results of a Mixed-Methods Study. Int J of Environ Res Public Health. 2021;18(24):13185. doi: 10.3390/ijerph182413185 34948792 PMC8701113

[52] Stevenson FA, Kerr C, Murray E, Nazareth I. Information from the Internet and the doctor-patient relationship: the patient perspective–a qualitative study. BMC Family Practice. 2007 Aug;8(1):47. doi: 10.1186/1471-2296-8-47 17705836 PMC2041946

[53] Tong A, Sainsbury P, Craig J. Consolidated criteria for reporting qualitative research (COREQ): a 32-item checklist for interviews and focus groups. Int J Qual Health Care. 2007 Dec;19(6):349–57. doi: 10.1093/intqhc/mzm042 17872937

[54] Kvale S, Brinkmann S. InterViews: Learning the craft of qualitative research interviewing, 2nd ed. Thousand Oaks, CA, US: Sage Publications, Inc; 2009. xviii, 354–xviii.

[55] British Association of Spine Surgeons. Cervical Stenosis and Myelopathy: Surgical Options. November 2018. Review date: October 2021. Available from: https://www.spinecompany.co.uk/pdf/cervical-stenosis-and-myelopathy-surgical-options-bass.pdf

[56] North Bristol NHS Trust. Information for patients undergoing Cervical (neck) surgery (Anterior and Posterior Approach). In: Service NH, editor. 2019. Available from: https://www.nbt.nhs.uk/sites/default/files/attachments/Information%20for%20patients%20undergoing%20Cervical%20%28neck%29%20surgery%20%28Anterior%20and%20Posterior%20Approach%29_NBT002412.pdf

[57] Cambridgeshire Community Services NHS Trust. Cervical Myelopathy information sheet including warning signs. 2020. Available from: https://www.eoemskservice.nhs.uk/docs/default-source/msk-condition-leaflets/cervical-myelopathy-information-sheet.pdf?sfvrsn=1bd99656_2

[58] Hilton B, Tempest-Mitchell J, Davies B, Kotter M. Assessment of degenerative cervical myelopathy differs between specialists and may influence time to diagnosis and clinical outcomes. PloS one. 2018;13(12):e0207709–e. doi: 10.1371/journal.pone.0207709 30557368 PMC6296508

[59] Fehlings MG, Tetreault LA, Riew KD, Middleton JW, Aarabi B, Arnold PM, et al. A Clinical Practice Guideline for the Management of Patients With Degenerative Cervical Myelopathy: Recommendations for Patients With Mild, Moderate, and Severe Disease and Nonmyelopathic Patients With Evidence of Cord Compression. Global Spine J. 2017 Sep;7(3 Suppl):70s–83s. doi: 10.1177/2192568217701914 29164035 PMC5684840

[60] Badhiwala JH, Ahuja CS, Akbar MA, Witiw CD, Nassiri F, Furlan JC, et al. Degenerative cervical myelopathy—update and future directions. Nat Rev Neurol. 2020 Feb;16(2):108–24. doi: 10.1038/s41582-019-0303-0 31974455

[61] Lannon M, Kachur E. Degenerative Cervical Myelopathy: Clinical Presentation, Assessment, and Natural History. J Clin Med. 2021 Aug 17;10(16):3626. doi: 10.3390/jcm10163626 34441921 PMC8396963

[62] Davies BM, Munro C, Khan DZ, Fitzpatrick SM, Hilton B, Mowforth OD, et al. Outcomes of Degenerative Cervical Myelopathy From The Perspective of Persons Living With the Condition: Findings of a Semistructured Interview Process With Partnered Internet Survey. Global Spine J. 2022 Apr;12(3):432–40. doi: 10.1177/2192568220953811 33203262 PMC9121154

[63] Macht M, Gerlich C, Ellgring H., Infopark Collaboration. Information Needs in Older Persons with Parkinson’s Disease in Germany: A Qualitative Study. Forum Qual Soc Res. 2003 Jan;4(1). doi: 10.17169/fqs-4.1.760

[64] Charles C, Gafni A, Whelan T. Decision-making in the physician–patient encounter: revisiting the shared treatment decision-making model. Soc Sci & Med. 1999 Sep;49(5):651–61. doi: 10.1016/s0277-9536(99)00145-8 10452420

[65] Sturges JE, Hanrahan KJ. Comparing Telephone and Face-to-Face Qualitative Interviewing: a Research Note. Qual Res. 2004;4(1):107–18. doi: 10.1177/1468794104041110

[66] Braun V, Clarke V. Using thematic analysis in psychology. Qual Res Psychol. 2006;3(2):77–101. doi: 10.1191/1478088706qp063oa

[67] Kiger ME, Varpio L. Thematic analysis of qualitative data: AMEE Guide No. 131. Med Teach. 2020 Aug;42(8):846–54. doi: 10.1080/0142159X.2020.1755030 32356468

[68] Vaismoradi M, Turunen H, Bondas T. Content analysis and thematic analysis: Implications for conducting a qualitative descriptive study. Nurs Health Sci. 2013 Sep;15(3):398–405; doi: 10.1111/nhs.12048 23480423

[69] Tuckett AG. Applying thematic analysis theory to practice: a researcher’s experience. Contemp Nurse. 2005 Jul-Aug;19(1–2):75–87. doi: 10.5172/conu.19.1-2.75 16167437

[70] Ose SO. Using Excel and Word to Structure Qualitative Data. J Appl Soc Sci. 2016;10(2):147–62. doi: 10.1177/1936724416664948

[71] Meyer DZ, Avery LM. Excel as a Qualitative Data Analysis Tool. Field Methods. 2008;21(1):91–112. doi: 10.1177/1525822X08323985

[72] Swallow V, Newton J, Van Lottum C. How to manage and display qualitative data using ‘Framework’ and Microsoft® Excel. J Clin Nurs. 2003 Jul;12(4):610–2. doi: 10.1046/j.1365-2702.2003.00728.x 12790875

[73] Bree RT, Gallagher G. Using Microsoft Excel to code and thematically analyse qualitative data: a simple, cost-effective approach. AISHE-J. 2016;8(2):2811–14.

[74] Frith H, Gleeson K. Clothing and embodiment: men managing body image and appearance. Psychol Men Masc. 2004;5(1):40–8. doi: 10.1037/1524-9220.5.1.40

[75] DiCicco-Bloom B, Crabtree BF. The qualitative research interview. Med Educ. 2006 Apr;40(4):314–21. doi: 10.1111/j.1365-2929.2006.02418.x 16573666

[76] Tiwary A, Rimal A, Paudyal B, Sigdel KR, Basnyat B. Poor communication by health care professionals may lead to life-threatening complications: examples from two case reports. Wellcome Open Res. 2019;4:7. doi: 10.12688/wellcomeopenres.15042.1 31448336 PMC6694717

[77] Davies BM, Khan DZ, Barzangi K, Ali A, Mowforth OD, Nouri A, et al. We Choose to Call it ‘Degenerative Cervical Myelopathy’: Findings of AO Spine RECODE-DCM, an International and Multi-Stakeholder Partnership to Agree a Standard Unifying Term and Definition for a Disease. Global Spine J. 2022 Jun; 29(21925682221111780). doi: 10.1177/21925682221111780 35769029 PMC10802519

[78] Ahuja CS, Badhiwala JH, Fehlings MG. “Time is spine”: the importance of early intervention for traumatic spinal cord injury. Spinal Cord. 2020;58(9):1037–9. doi: 10.1038/s41393-020-0477-8 32393795 PMC7471096

[79] Russell BJ, Ward AM. Deciding what information is necessary: do patients with advanced cancer want to know all the details? Cancer Manag Res. 2011;3:191–9. doi: 10.2147/CMR.S12998 21792328 PMC3139480

[80] Kao MH, Tsai YF. Illness experiences in middle-aged adults with early-stage knee osteoarthritis: findings from a qualitative study. J Adv Nurs. 2014;70(7):1564–72. doi: 10.1111/jan.12313 24237307

[81] Płotka A, Prokop E, Migaj J, Straburzyńska-Migaj E, Grajek S. Patients’ knowledge of heart failure and their perception of the disease. Patient Prefer Adherence. 2017;11:1459–67. doi: 10.2147/PPA.S126133 28883713 PMC5576702

[82] Jankowska-Polańska B, Uchmanowicz I, Dudek K, Mazur G. Relationship between patients’ knowledge and medication adherence among patients with hypertension. Patient preference and adherence. 2016;10:2437–47. doi: 10.2147/PPA.S117269 27994443 PMC5153315

[83] Sweileh WM, Zyoud SH, Abu Nab’a RJ, Deleq MI, Enaia MI, Nassar SM, et al. Influence of patients’ disease knowledge and beliefs about medicines on medication adherence: findings from a cross-sectional survey among patients with type 2 diabetes mellitus in Palestine. BMC Public Health. 2014;14:94. doi: 10.1186/1471-2458-14-94 24479638 PMC3909379

[84] Skiveren J, Philipsen P, Therming G. Patients with psoriasis have insufficient knowledge of their risk of atherothrombotic disease and metabolic syndrome. Clin Exp Dermatol. 2015;40(6):600–4. doi: 10.1111/ced.12628 25847319

[85] Mäkeläinen P, Vehviläinen-Julkunen K, Pietilä AM. Rheumatoid arthritis patients’ knowledge of the disease and its treatments: a descriptive study. Musculoskeletal Care. 2009 Mar;7(1):31–44. doi: 10.1002/msc.138 18697184

[86] Legido-Quigley H, Camacho Lopez PA, Balabanova D, Perel P, Lopez-Jaramillo P, Nieuwlaat R, et al. Patients’ knowledge, attitudes, behaviour and health care experiences on the prevention, detection, management and control of hypertension in Colombia: a qualitative study. PLoS One. 2015;10(4):e0122112. doi: 10.1371/journal.pone.0122112 25909595 PMC4409332

[87] Patients’ knowledge of their chronic disease. Australian Journal for General Practitioners. 2013;42:411–6. 23781550

[88] Robinson A, Thompson DG, Wilkin D, Roberts C., Northwest Gastrointestinal Research Group. Guided self-management and patient-directed follow-up of ulcerative colitis: a randomised trial. Lancet. 2001;358(9286):976–81. doi: 10.1016/s0140-6736(01)06105-0 11583752

[89] Newcomb PA, McGrath KW, Covington JK, Lazarus SC, Janson SL. Barriers to Patient-Clinician Collaboration in Asthma Management: The Patient Experience. J Asthma. 2010 Mar;47(2):192–7. doi: 10.3109/02770900903486397 20170328

[90] Schroeder SA. Shattuck Lecture. We can do better—improving the health of the American people. N Engl J Med. 2007;357(12):1221–8. doi: 10.1056/NEJMsa073350 17881753

[91] U.S. Department of Health & Human Services. Multiple chronic conditions: A Strategic Framework: Optimum Health and Quality of Life for Individuals with Multiple Chronic Conditions. Washington, DC: 2010.

[92] National Health Service England. The NHS belongs to the people: a call to action. London: 2013. Available from: https://www.england.nhs.uk/wp-content/uploads/2013/07/nhs-belongs.pdf

[93] Newton P, Asimakopoulou K, Scambler S. Information seeking and use amongst people living with type 2 diabetes: an information continuum. Int J Health Promot and Educ. 2012;50(2):92–9. doi: 10.1080/14635240.2012.665581

[94] Huskamp HA, Keating NL, Malin JL, Zaslavsky AM, Weeks JC, Earle CC, et al. Discussions with physicians about hospice among patients with metastatic lung cancer. Arch Intern Med. 2009;169(10):954–62. doi: 10.1001/archinternmed.2009.127 19468089 PMC2689617

[95] Temel JS, Greer JA, Admane S, Gallagher ER, Jackson VA, Lynch TJ, et al. Longitudinal Perceptions of Prognosis and Goals of Therapy in Patients With Metastatic Non–Small-Cell Lung Cancer: Results of a Randomized Study of Early Palliative Care. Journal of Clinical Oncology. 2011;29(17):2319–26. doi: 10.1200/JCO.2010.32.4459 21555700

[96] Holloway RG, Gramling R, Kelly AG. Estimating and communicating prognosis in advanced neurologic disease. Neurology. 2013;80(8):764–72. doi: 10.1212/WNL.0b013e318282509c 23420894 PMC3589298

[97] Weeks JC, Catalano PJ, Cronin A, Finkelman MD, Mack JW, Keating NL, et al. Patients’ Expectations about Effects of Chemotherapy for Advanced Cancer. New England Journal of Medicine. 2012;367(17):1616–25 doi: 10.1056/NEJMoa1204410 23094723 PMC3613151

[98] Ahalt C, Walter LC, Yourman L, Eng C, Pérez-Stable EJ, Smith AK. "Knowing is better": preferences of diverse older adults for discussing prognosis. Journal of general internal medicine. 2012;27(5):568–75 doi: 10.1007/s11606-011-1933-0 22127798 PMC3326105

[99] Sullivan R, Ugalde A, Sinclair C, Breen LJ. Developing a Research Agenda for Adult Palliative Care: A Modified Delphi Study. J Palliat Med. 2019;22(5):480–8 doi: 10.1089/jpm.2018.0462 30461347

[100] Nwodoh CO, Okoronkwo IL, Nwaneri AC, Ndubuisi I, Chinweuba AU, Iheanacho P. Terminally-ill patients’ prognosis information preferences in an African setting: A qualitative descriptive study. International Journal of Africa Nursing Sciences. 2020;13:100220 10.1016/j.ijans.2020.100220

[101] Buckman R. Breaking bad news: why is it still so difficult? British Medical Journal (Clinical research ed). 1984;288(6430):1597–9 doi: 10.1136/bmj.288.6430.1597 6426658 PMC1441225

[102] Christakis NA, Iwashyna TJ. Attitude and self-reported practice regarding prognostication in a national sample of internists. Arch Intern Med. 1998;158(21):2389–95 doi: 10.1001/archinte.158.21.2389 9827791

[103] Rabow MW, McPhee SJ. Beyond breaking bad news: how to help patients who suffer. West J Med. 1999;171(4):260–3. 10578682 PMC1305864

[104] Fried TR, Bradley EH, O’Leary J. Prognosis communication in serious illness: perceptions of older patients, caregivers, and clinicians. J Am Geriatr Soc. 2003 Oct;51(10):1398–403. doi: 10.1046/j.1532-5415.2003.51457.x 14511159

[105] Jenkins V, Fallowfield L, Saul J. Information needs of patients with cancer: results from a large study in UK cancer centres. Br J Cancer. 2001;84(1):48–51. doi: 10.1054/bjoc.2000.1573 11139312 PMC2363610

[106] Clayton JM, Butow PN, Tattersall MH. When and how to initiate discussion about prognosis and end-of-life issues with terminally ill patients. J Pain Symptom Manage. 2005 Aug;30(2):132–44. doi: 10.1016/j.jpainsymman.2005.02.014 16125028

[107] Hagerty RG, Butow PN, Ellis PA, Lobb EA, Pendlebury S, Leighl N, et al. Cancer patient preferences for communication of prognosis in the metastatic setting. J Clin Oncol. 2004;22(9):1721–30. doi: 10.1200/JCO.2004.04.095 15117995

[108] Kirshblum S, Fichtenbaum J. Breaking the news in spinal cord injury. J Spinal Cord Med. 2008;31(1):7–12. doi: 10.1080/10790268.2008.11753975 18533406 PMC2435037

[109] Leydon GM, Boulton M, Moynihan C, Jones A, Mossman J, Boudioni M, et al. Cancer patients’ information needs and information seeking behaviour: in depth interview study. BMJ. 2000;320(7239):909–13. doi: 10.1136/bmj.320.7239.909 10742000 PMC27332

[110] Jansen J, van Weert J, van Dulmen S, Heeren T, Bensing J. Patient education about treatment in cancer care: an overview of the literature on older patients’ needs. Cancer Nurs. 2007;30(4):251–60. doi: 10.1097/01.NCC.0000281735.25609.af 17666973

[111] Nicolaije KA, Husson O, Ezendam NP, Vos MC, Kruitwagen RF, Lybeert ML, et al. Endometrial cancer survivors are unsatisfied with received information about diagnosis, treatment and follow-up: a study from the population-based PROFILES registry. Patient Educ Couns. 2012 Sep;88(3):427–35. doi: 10.1016/j.pec.2012.05.002 22658248

[112] Woodcock H, Gillam S. ’A one-to-one thing is better than a thousand books’: views and understanding of older people with diabetes. Qual Prim Care. 2013;21(3):157–63. 23968265

[113] Lesnovska KP, Börjeson S, Hjortswang H, Frisman GH. What do patients need to know? Living with inflammatory bowel disease. J Clin Nurs. 2014 Jun;23(11–12):1718–25. doi: 10.1111/jocn.12321 24004406

[114] Back AL, Arnold RM. Discussing Prognosis: “How Much Do You Want to Know?” Talking to Patients Who Do Not Want Information or Who Are Ambivalent. J Clin Oncol. 2006;24(25):4214–7. doi: 10.1200/JCO.2006.06.008 16943540

[115] Nunstedt H RG, Alsen P et al. Patients´ Variations of Reflection About and Understanding of Long-Term Illness- Impact of Illness Perception on Trust in Oneself or Others. Open Nurs J 2017;11(1):43–53. doi: 10.2174/1874434601711010043 28567169 PMC5420171

[116] Megari K. Quality of Life in Chronic Disease Patients. Health Psychol Res. 2013;1(3):e27. doi: 10.4081/hpr.2013.e27 26973912 PMC4768563

[117] Kopjar B, Bohm PE, Arnold JH, Fehlings MG, Tetreault LA, Arnold PM. Outcomes of Surgical Decompression in Patients With Very Severe Degenerative Cervical Myelopathy. Spine (Phila Pa 1976). 2018 Aug;43(16):1102–9. doi: 10.1097/BRS.0000000000002602 29462066 PMC6066419

[118] Brody DS, Miller SM, Lerman CE, Smith DG, Caputo GC. Patient perception of involvement in medical care: relationship to illness attitudes and outcomes. J Gen Intern Med. 1989 Nov-Dec;4(6):506–11. doi: 10.1007/BF02599549 2585158

[119] Cassileth BR, Zupkis RV, Sutton-Smith K, March V. Information and participation preferences among cancer patients. Ann Intern Med. 1980 Jun;92(6):832–6. doi: 10.7326/0003-4819-92-6-832 7387025

[120] Gobbo M, Saldaña R, Rodríguez M, Jiménez J, García-Vega MI, de Pedro JM, et al. Patients’ Experience and Needs During Perioperative Care: A Focus Group Study. Patient Prefer Adherence. 2020;14:891–902. doi: 10.2147/PPA.S252670 32546983 PMC7266520

[121] Larsson IE, Sahlsten MJ, Segesten K, Plos KA. Patients’ perceptions of barriers for participation in nursing care. Scand J Caring Sci. 2011;25(3):575–82. doi: 10.1111/j.1471-6712.2010.00866.x 21241347

[122] Chen H-H, Yeh M-L, Yang H-J. Testing the impact of a multimedia video CD of patient-controlled analgesia on pain knowledge and pain relief in patients receiving surgery. Int J Med Inform. 2005 Jul;74(6):437–45. doi: 10.1016/j.ijmedinf.2005.04.003 15936246

[123] Davies BM, Munro CF, Kotter MR. A Novel Insight Into the Challenges of Diagnosing Degenerative Cervical Myelopathy Using Web-Based Symptom Checkers. J Med Internet Res. 2019;21(1):e10868–e. doi: 10.2196/10868 30300137 PMC6330198

[124] Carlin LE, Smith HE, Henwood F. To see or not to see: a qualitative interview study of patients’ views on their own diagnostic images. BMJ Open. 2014;4(7):e004999–e doi: 10.1136/bmjopen-2014-004999 25082418 PMC4120403

[125] Petersen LL, Birkelund R, Ammentorp J, Schiøttz-Christensen B. “An MRI reveals the truth about my back”: a qualitative study about patients’ expectations and attitudes toward the value of MRI in the assessment of back pain. Eur J Pers Cent Healthc. 2016;5:453–8. doi: 10.5750/ejpch.v4i3.1122

[126] Williams R, Murray A. Prevalence of depression after spinal cord injury: a meta-analysis. Arch Phys Med Rehabil. 2015 Jan;96(1):133–40. doi: 10.1016/j.apmr.2014.08.016 25220943

[127] Davies BM, Mowforth O, Wood H, Karimi Z, Sadler I, Tetreault L, et al. Improving Awareness Could Transform Outcomes in Degenerative Cervical Myelopathy [AO Spine RECODE-DCM Research Priority Number 1]. Global Spine J. 2022 Feb;12(1_suppl):28S–38S. doi: 10.1177/21925682211050927 35174734 PMC8859708

[128] Baird CL. Holding on self-caring with osteoarthritis. J Gerontol Nurs. 2003;29(6):32–9. doi: 10.3928/0098-9134-20030601-08 12830654

[129] Walsh MC, Trentham-Dietz A, Schroepfer TA, Reding DJ, Campbell B, Foote ML, et al. Cancer Information Sources Used by Patients to Inform and Influence Treatment Decisions. J Health Commun. 2010 Jun;15(4):445–63. doi: 10.1080/10810731003753109 20574881

[130] Magnezi R, Bergman YS, Grosberg D. Online activity and participation in treatment affects the perceived efficacy of social health networks among patients with chronic illness. J Med Internet Res. 2014;16(1):e12. doi: 10.2196/jmir.2630 24413148 PMC3906665

[131] Kim J-N, Lee S. Communication and Cybercoping: Coping With Chronic Illness Through Communicative Action in Online Support Networks. J Health Commun. 2014;19(7):775–94. doi: 10.1080/10810730.2013.864724 24559492

[132] Rogers A, Vassilev I, Sanders C, Kirk S, Chew-Graham C, Kennedy A, et al. Social networks, work and network-based resources for the management of long-term conditions: a framework and study protocol for developing self-care support. Implement Sci. 2011;6:56. doi: 10.1186/1748-5908-6-56 21619695 PMC3120720

[133] Ziebland S, Wyke S. Health and illness in a connected world: how might sharing experiences on the internet affect people’s health? Milbank Q. 2012;90(2):219–49. doi: 10.1111/j.1468-0009.2012.00662.x 22709387 PMC3460203

[134] Vassilev I, Rogers A, Kennedy A, Koetsenruijter J. The influence of social networks on self-management support: a metasynthesis. BMC Public Health. 2014;14:719. doi: 10.1186/1471-2458-14-719 25023948 PMC4223639

[135] Allen C, Vassilev I, Kennedy A, Rogers A. Long-Term Condition Self-Management Support in Online Communities: A Meta-Synthesis of Qualitative Papers. J Medical Internet Res. 2016;18(3):e61–e. doi: 10.2196/jmir.5260 26965990 PMC4807245

[136] Moreno-Serra R, Anaya-Montes M, León-Giraldo S, Bernal O. Addressing recall bias in (post-)conflict data collection and analysis: lessons from a large-scale health survey in Colombia. Conflict Health. 2022;16(1):14. doi: 10.1186/s13031-022-00446-0 35395772 PMC8994310

[137] National Health Service England. Involving people in their own health and care: Statutory guidance for clinical commissioning groups and NHS England. Available from: https://www.england.nhs.uk/wp-content/uploads/2017/04/ppp-involving-people-health-care-guidance.pdf

[138] Main BG, McNair AGK, Huxtable R, Donovan JL, Thomas SJ, Kinnersley P et al.Core information sets for informed consent to surgical interventions: baseline information of importance to patients and clinicians. BMC Med Ethics. 2017;18(1). doi: 10.1186/s12910-017-0188-7 28446164 PMC5406972

[139] Main BG, McNair AGK, Haworth S, Rooshenas L, Hughes CW, Tierney P, et al. Core information set for informed consent to surgery for oral or oropharyngeal cancer: A mixed-methods study. Clin Otolaryngol. 2018;43(2):624–31. doi: 10.1111/coa.13037 29178168

[140] Blazeby JM, Macefield R, Blencowe NS, Jacobs M, McNair AGK, Sprangers M, et al. Core information set for oesophageal cancer surgery. Br J Surg. 2015 Jul;102(8):936–43. doi: 10.1002/bjs.9840 25980524

[141] McNair AGK, Whistance RN, Main B, Forsythe R, Macefield R, Rees J, et al. Development of a core information set for colorectal cancer surgery: a consensus study. BMJ Open. 2019;9(11):e028623. doi: 10.1136/bmjopen-2018-028623 31727644 PMC6886994

[142] Tariman JD, Doorenbos A, Schepp KG, Singhal S, Berry DL. Information Needs Priorities in Patients Diagnosed With Cancer: A Systematic Review. J Adv Pract Oncol. 2014;2014(5):115–22. 24910808 PMC4042668

