# Supplementary material for: The information needs of people with degenerative cervical myelopathy: A qualitative study to inform patient education in clinical practice
Source: PLoS One. 2023 May 19;18(5):e0285334. doi: 10.1371/journal.pone.0285334 (PMC10198551; doi:10.1371/journal.pone.0285334)
Supplement: S1 Appendix — (DOCX) [file pone.0285334.s001.docx]

**Appendix**

**INTERVIEW SCHEDULE**

**INTRODUCTION**

- Study aim
- Procedure
- Estimated duration
- Confidentiality and anonymity
- Participation is entirely voluntary
- Request for permission to audio record the interview

**ONSET OF SYMPTOMS**

Given the knowledge and information you had at the time, what did you think was happening when you first noticed your DCM symptoms?

**DIAGNOSIS & TREATMENT**

What information did you receive at diagnosis?

- What information, if any, did you receive about the condition? What information would you have liked to have?
- What information, if any, did you receive about the prognosis of this condition? What information would you have liked to have?
- What information, if any, did you receive about the surgical treatment? What information would you have liked to have?
- What were you advised to do while waiting for surgery? What information would you have liked to have?
- Which information, if any, was useful? Why?
- Was there any other information you would have wanted to have?

**POST-TREATMENT MANAGEMENT & LONG-TERM MANAGEMENT**

What information did you receive regarding the post-treatment management of DCM?

- What were you advised to do after surgery? What information would you have liked to have?
- What information, if any, did you receive about the long-term management of DCM? What information would you have liked to have?
- What information, if any, did you receive about the financial or mental-health implications of living with DCM? What information would you have liked to have?
- Which information, if any, was useful? Why?
- Was there any other information you would have wanted to have?
