# Supplementary material for: The information needs of people with degenerative cervical myelopathy: A qualitative study to inform patient education in clinical practice
Source: PLoS One. 2023 May 19;18(5):e0285334. doi: 10.1371/journal.pone.0285334 (PMC10198551; doi:10.1371/journal.pone.0285334)
Supplement: S1 Table — (DOCX) [file pone.0285334.s002.docx]

**Table 1**: Characteristics of Interview Participants

| Characteristic |  | Frequency  n (%) |
| --- | --- | --- |
| Gender | Female | 13 (65%) |
|  | Male | 7 (35%) |
| Age Range | 31 – 40 | 1 (5%) |
|  | 41 - 50 | 2 (10%) |
|  | 51 – 60 | 9 (45%) |
|  | 61 – 70 | 6 (30%) |
|  | 71 – 80 | 2 (10%) |
| Country | Canada | 2 (10%) |
|  | United Kingdom | 15 (75%) |
|  | United States | 3 (15%) |
| Employment Status | Employed | 8 (40%) |
|  | Unemployed* | 5 (25%) |
|  | Retired | 7 (35%) |
| Years Since Diagnosis | 0 – 2 years | 12 (60%) |
|  | 3 – 10 years | 7 (35%) |
|  | 10+ | 1 (5%) |
| DCM Surgery | Yes | 18 (90%) |
|  | No | 2 (10%) |
| Treatment | ACDF | 14 (70%) |
|  | Laminectomy | 3 (15%) |
|  | Corpectomy | 1 (10%) |
|  | Nonoperative Management | 2 (10%) |

*Unable to work due to disability

ACDF: anterior cervical discectomy and fusion
