# Supplementary material for: The information needs of people with degenerative cervical myelopathy: A qualitative study to inform patient education in clinical practice
Source: PLoS One. 2023 May 19;18(5):e0285334. doi: 10.1371/journal.pone.0285334 (PMC10198551; doi:10.1371/journal.pone.0285334)
Supplement: S2 Table — (DOCX) [file pone.0285334.s003.docx]

**Table 2.** **Main Themes and Subthemes**

| **Global Themes** | **Subthemes** |
| --- | --- |
| **Variations in the provision of information during clinical interactions** | - Pathophysiology, symptomatology, clinical course, and impact on quality of life |
|  | - The surgical treatment |
|  |  |
| **Variations in the information needs of PwCM** | - The negative implications of lacking awareness of DCM |
|  | - Pathophysiology, symptomatology, clinical course, impact on quality of life |
|  | - The surgical treatment |
|  | - Adjusting to life with disability |
|  |  |
| **Information items PwCM find useful** | - The MRI as a pedagogic tool |
|  | - Varied useful information items |
|  | - The Support Group as an information source |
